# Experimental playback of urban noise does not affect cognitive performance in captive Australian magpies

**DOI:** 10.1242/bio.060535

**Published:** 2024-08-14

**Authors:** Farley Connelly, Robin D. Johnsson, Raoul A. Mulder, Michelle L. Hall, John A. Lesku

**Affiliations:** ^1^ School of BioSciences, The University of Melbourne, Melbourne, Victoria 3010, Australia; ^2^ School of Agriculture, Biomedicine and Environment, La Trobe University, Melbourne, Victoria 3086, Australia; ^3^ Bush Heritage Australia, Melbourne, Victoria 3000, Australia; ^4^ School of Biological Sciences, The University of Western Australia, Perth, Western Australia 6009, Australia; ^5^ Research Centre for Future Landscapes, La Trobe University, Melbourne, Victoria 3086, Australia; ^6^ Alameda County Resource Conservation District, Livermore, California 94550, USA; ^7^ Department of Psychology, Franklin and Marshall College, Lancaster, Pennsylvania 17603, USA

**Keywords:** Anthropogenic noise, Behavioural flexibility, Birds, Inhibitory control, Learning, Noise pollution, Reversal learning, Spatial learning

## Abstract

Exposure of wildlife to anthropogenic noise is associated with disruptive effects. Research on this topic has focused on behavioural and physiological responses of animals to noise, with little work investigating links to cognitive function. Neurological processes that maintain cognitive performance can be impacted by stress and sleep disturbances. While sleep loss impairs cognitive performance in Australian magpies, it is unclear whether urban noise, which disrupts sleep, can impact cognition as well. To fill this gap, we explored how environmentally relevant urban noise affected the performance of wild-caught, city-living Australian magpies (*Gymnorhina tibicen tyrannica*) on a cognitive task battery including associative and reversal learning, inhibitory control, and spatial memory. Birds were housed and tested in a laboratory environment; sample sizes varied across tasks (*n*=7–9 birds). Tests were conducted over 4 weeks, during which all magpies were exposed to both an urban noise playback and a quiet control. Birds were presented with the entire test battery twice: following exposure to, and in the absence of, an anthropogenic noise playback; however, tests were always performed without noise (playback muted during testing). Magpies performed similarly in both treatments on all four tasks. We also found that prior experience with the associative learning task had a strong effect on performance, with birds performing better on their second round of trials. Like previous findings on Australian magpies tested on the same tasks in the wild under noisy conditions, we could not find any disruptive effects on cognitive performance in a controlled experimental laboratory setting.

## Teaser

**Summary:** This study investigates the impact of urban noise on bird cognitive performance. Despite disrupting sleep, noise exposure did not affect cognitive performance, revealing insights into wildlife adaptation to anthropogenic noise.

## INTRODUCTION

Exposure to human-generated (or anthropogenic) noise is an important conservation issue for a diverse range of species (Kunc and Schmidt, 2019). For example, in the United States over 80% of all land, including some of the most protected and ecologically sensitive areas, is within 1 km of a road and its associated traffic noise (Buxton et al., 2017). Even more remote areas may be affected by the soundscape generated by overhead flight paths of aircraft (Buxton et al., 2019).

There is increasing evidence for negative impacts of noise on animals in both terrestrial and aquatic habitats (Duarte et al., 2021; Kunc and Schmidt, 2019). Noise has been implicated in reduced breeding success in brown-headed nuthatches (*Sitta pusilla*; Ernstes and Quinn, 2016) and zebra finches (*Taeniopygia guttata*; Potvin and MacDougall-Shackleton, 2015); behavioural changes of migrating bird species (McClure et al., 2013, 2017); and decreased survival in great tits (*Parus major*; Raap et al., 2017), house sparrows (*Passer domesticus;* Schroeder et al., 2012), and other wildlife species (Francis and Barber, 2013; Kight and Swaddle, 2011). Furthermore, changes to the frequency and timing of vocalizations have been observed in European robins (*Erithacus rubecula*; Fuller et al., 2007), spotted doves (*Streptopelia chinensis*; Guo et al., 2016), eastern bluebirds (*Sialia sialis*; Kight and Swaddle, 2015), and silvereyes (*Zosterops lateralis*; Potvin et al., 2014), and changes in anti-predator behaviours have been seen in diverse taxa (Barber et al., 2010), including house sparrows (Meillère et al., 2015) and peahens (*Pavo cristatus*; Yorzinski et al., 2015).

Relatively little is known about the impact of noise pollution on cognitive performance outside of humans and rodents. Studies on humans have shown that acute exposure to anthropogenic noise impairs attention in children, leading to reduced comprehension and spatial memory; noise (at night) also disrupts sleep, leading to reduced cognitive performance the following day (Alhola and Polo-Kantola, 2007; Barzegar et al., 2015; Elmenhorst et al., 2012; Halperin, 2014; Kabadayi et al., 2018; Smith, 2012; Stansfeld and Clark, 2015; Szalma and Hancock, 2011). Similar findings have been reported in mice and rats, with acute exposure to noise affecting spatial memory (Hu et al., 2014), and chronic exposure impacting memory and sleep following the removal of noise (Jafari et al., 2018; Rabat et al., 2006). Still, some research on birds shows that anthropogenic noise impairs cognitive performance, including motor learning, inhibitory control, spatial memory, and social learning (Daria et al., 2023; Evans et al., 2018; Osbrink et al., 2021). Perhaps surprisingly, urban noise also affects bill colour, making bills less bright or turning them redder, which has important consequences for social interactions and mate choice (Daria et al., 2023). Furthermore, noise can also make it more difficult for black-capped chickadees *Poecile atricapillus* to learn to discriminate conspecific songs (Montenegro et al., 2021; Osbrink et al., 2021).

Importantly, urban noise has a disruptive effect on sleep in Australian magpies *Gymnorhina tibicen tyrannica* (Connelly et al., 2020). Specifically, magpies exposed to 24 h of anthropogenic noise in a controlled laboratory setting, spent less time asleep, and when they did sleep, sleep was more fragmented, sleep episodes were shorter, and non-rapid eye movement sleep was less intense (Connelly et al., 2020). In a second study, when the same individual magpies were kept awake via a gentle handling technique for an entire 12-h night, their performance only on a reversal learning task was impaired and characteristics and timing of their singing changed (Johnsson et al., 2022a). Given that urban noise disrupts sleep (Connelly et al., 2020), and sleep loss impairs cognition in Australian magpies (Johnsson et al., 2022a), we wanted to investigate whether urban noise could have stand-alone impacts on cognition. A recent study in Western Australia showed that wild Australian magpies (*Gymnorhina tibicen dorsalis*) decrease their foraging, vocalisations behaviours, and anti-predator responses in the presence of high levels of anthropogenic noise (Blackburn et al., 2023); however, a previous study found no evidence for noise-mediated cognitive impairments in wild Australian magpies in Victoria (*Gymnorhina tibicen tyrannica*; Connelly et al., 2022) and relatively little is known about impacts in other bird species. Therefore, a controlled laboratory setting may be more insightful than wild studies in which extraneous sources of variation abound.

Measuring variation in cognitive performance in wild animals is challenging (Griffin et al., 2015; Morand-Ferron et al., 2016; Shaw, 2017). Individuals must be identifiable, and ideally have fidelity to a particular site. Those animals must then be trained to perform the cognitive task(s) and, once trained, must be motivated to perform repeatedly, thus limiting both the tests that can be employed and the species that can be studied. Traditionally, cognitive studies on animals have focused on a single cognitive feature, such as tool use or spatial memory (Emery and Clayton, 2004). While such studies demonstrate that some animals are capable of impressive cognitive feats, they are less useful as behavioural assays of an animal's complement of cognitive abilities (Shaw, 2017). In addition, performance on any one cognitive task can be influenced by experience, motivation, and/or ecological relevance of the task per se. To quantify cognitive ability more completely, researchers have developed batteries of cognitive tests that allow for the evaluation of performance in multiple cognitive domains. Such tests are increasingly being utilized for wildlife, notably birds (Ashton et al., 2018; Blackburn et al., 2022; Boogert et al., 2011; Connelly et al., 2022; Isden et al., 2013; Shaw et al., 2015).

To understand what effect prolonged noise exposure might have on cognitive performance in a controlled environment, we brought wild Australian magpies into captivity and compared the cognitive performance of individual birds following prolonged exposure to, and in the absence of, an urban noise recording, using an established cognitive test battery (Ashton et al., 2018; Connelly et al., 2022). Australian magpies are large passerines of the family Artamidae. They are widely abundant across both rural and urban habitats of Australia, conspicuous inhabitants of cities, tolerate close interactions with humans, can easily be trained to appear when called, and are capable of completing cognitive tasks in the wild (Ashton et al., 2018; 2022; Connelly et al., 2022; Diquelou et al., 2016; Mirville et al., 2016; Rollinson and Jones, 2006; Sollis et al., 2023). Together, this makes Australian magpies a great model to test the effects of urbanization on several cognitive domains.

As we previously tested the impact of urban noise on cognitive performance in Australian magpies in the wild finding no effects (Connelly et al., 2022), we wanted to test magpies on the same cognitive tests, but in a controlled laboratory environment. If variation associated with a non-standardised testing environment in the wild was the reason why we did not detect any noise-related effects on cognition in the previous experiment (Connelly et al., 2022), then testing magpies in a controlled and standardised environment would unmask any such effects on cognitive performance.

## RESULTS

**Fig. 1.**
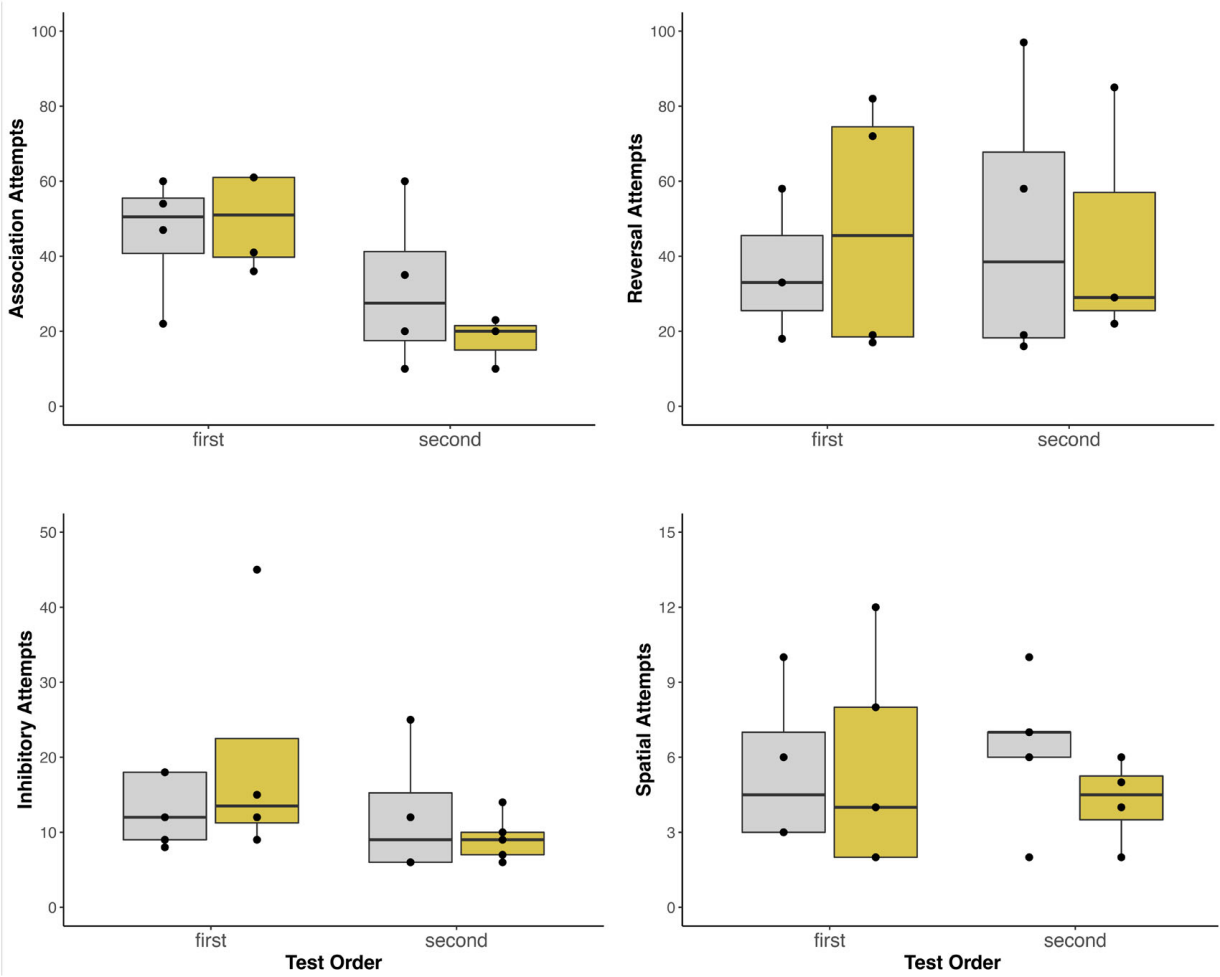
**Effect of urban noise on cognitive performance.** The number of trials required to solve each task: colour association, reversal learning, inhibitory control, and spatial memory. Scores are grouped by treatment (control: grey; noise: yellow) and test order (first or second).

We found no difference in performance across the battery of cognitive tasks under the noise condition compared to the control, suggesting that exposure to urban noise did not affect cognitive performance (Table 1, Fig. 1). Performance (number of attempts required to complete the task) also did not differ between the sexes in either treatment (average attempts: control: female, 4.50±2.38; male, 5.33±2.08; noise: female, 4.50±2.52; male, 6.33±5.13). The only noteworthy significant effect is that birds performed better on the associative learning when they attempted it a second time, independent of the acoustic environment (see Associative learning).

**Table 1.**
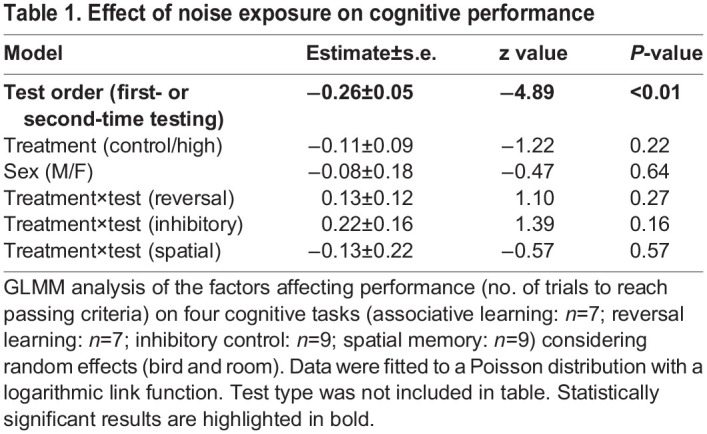
Effect of noise exposure on cognitive performance

### Associative learning

Individuals (*n*=7) required 36.3±20.0 trials to pass in the control treatment and 36.0±19.9 trials in the noise treatment (*t*=−0.03, *P*=0.98). The performance of all birds on the association task greatly improved on their second attempt, requiring almost one-half the number of attempts (first: 46.9±15.0 trials; second: 25.2±17.5 trials; *t*=−3.68, *P*=0.01).

### Reversal learning

Individuals (*n*=7) required 42.7±30.0 trials to pass in the control treatment and 46.6±31.5 trials in the noise treatment (*t*=0.24, *P*=0.82). Test order had little effect on the reversal task, with birds performing similarly on both test trials (first: 42.7±27.6 trials; second: 46.6±33.6 trials; *t*=0.24, *P*=0.82).

### Inhibitory control

Magpies (*n*=9) averaged 11.4±7.3 trials in the control treatment and 12.7±12.1 trials in the noise treatment. There was little variation between the performance of birds on the control tasks in either treatment (cylinder: 4.5±2.2 trials in control, 4.8±3.1 trials with noise; wall: 4.4±2.2 in control, 4.2±1.4 trials with noise). Scores between the two tasks in each treatment were not different (control: t=0.12, *P*=0.91; noise: *t*=0.60, *P*=0.56).

**Fig. 2.**
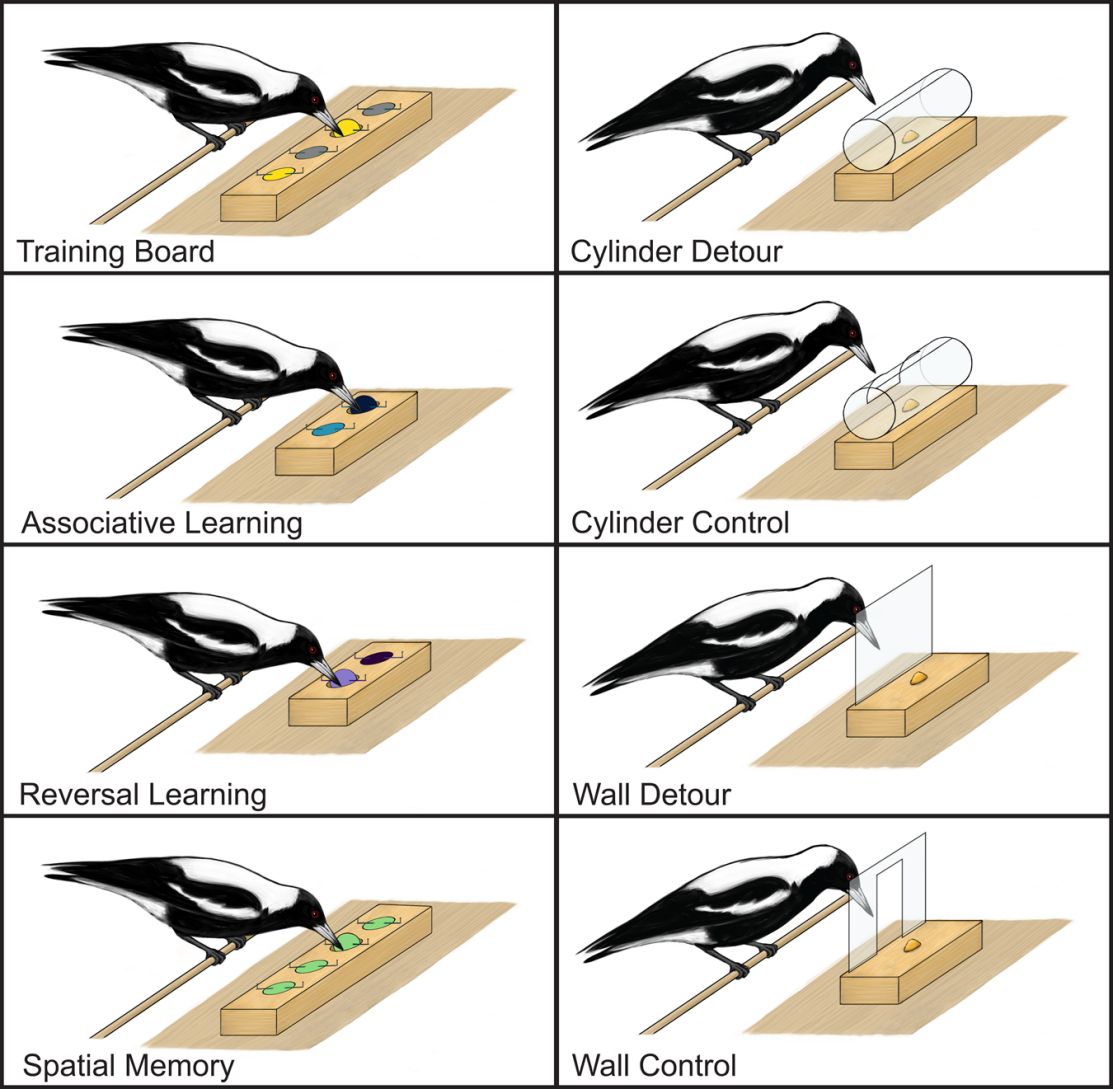
All test apparatuses used in the cognitive test battery including training, associative and reversal learning (purple and blue), spatial memory, and all inhibitory tasks: cylinder detour task, cylinder control task, wall detour task, and wall control task.

Independent of noise, birds successfully detoured around the cylinder 65% of the time and the wall task 75% of the time. In the control tasks (Fig. 2), birds retrieved the reward through the hole in the cylinder and wall 85% of the time.

### Spatial memory

Birds did not learn the spatial memory task and averaged 6.0±2.9 trials in the control and 5.0±3.3 in the noise treatment (*t*=0.34, *P*=0.74). For both the noise and control treatments, magpies selected wells at random (26% correct choices) after both 24 h and 48 h. Test order did not affect the spatial task, with birds performing equally on the task in both testing periods (first: 5.6±3.7 trials; second: 5.4±2.6 trials; *t*=−0.58, *P*=0.58). In the olfactory test, the likelihood of selecting the well that had contained food was not significantly different from selecting any other well (*t*=0.39, *P*=0.77), though the magpies did increase their performance to 42.9% correct choices from 26%.

## DISCUSSION

Across four cognitive tests that included associative and reversal learning, inhibitory control, and spatial memory, the performance of wild-caught Australian magpies was similar when exposed to chronic urban noise (outside of the testing period) or in the absence of the noise exposure. Overall, birds had slightly lower average scores in control than noise for two of the four tests, but no statistically significant effects of the noise treatment.

Performance on the associative learning task was significantly better on the second presentation regardless of the noise exposure. Despite being causally identical but visually distinct (different coloured caps), the magpies were able to learn the association task after repeated testing.

Associative and reversal learning, and inhibitory control was resilient to urban noise in captivity. This is consistent with results found in wild Victorian Australian magpies (Connelly et al., 2022); however, opposes what was observed in wild Western Australian subspecies and captive zebra finches (Osbrink et al., 2021). Unlike our captive magpies, zebra finches in Osbrink et al. (2021) were only exposed to noise during testing, suggesting that acute (or immediate) effects of noise have a greater impact than longer-term effects (Jafari et al., 2019; Klatte et al., 2013). Additionally, performance, including motivation, attention, and flexibility on a similar reversal learning task is dependent on the amount of prior sleep (Johnsson et al., 2022a). Given urban noise can restrict sleep in magpies (Connelly et al., 2020), the magnitude of noise-induced night-time wakefulness must be inconsequential for subsequent day-time performance. If true, this bodes well for the health and well-being of city-living Australian magpies.

When tested without noise despite prolonged noise exposure, cognitive performance of our magpies was similar to magpies that had not had any exposure to noise. Interestingly, studies in humans have shown that environmental noise exposure can impair cognitive performance, and that removing the noise restores performance (Clark et al., 2013; Stansfeld and Clark, 2015). Therefore, our experimental design may have removed the factor most influencing the observed changes in cognitive performance in the presence of noise. In an urban environment, noise is constant (although variable), and can affect birds both immediately (e.g. distraction causing lapses in attention) and over days (e.g. stress response, sleep loss). By silencing noise during testing, we sought to remove any immediate effects, and instead focused on sustained effects of long-term noise exposure on cognitive performance. This design meant we were unable to gauge any immediate effects of noise. There remains value in testing birds in the presence of acute noise to understand any immediate effects on cognitive performance.

Noise has been shown to produce a stress response in birds (Kleist et al., 2018; Wright et al., 2007), such that increasing corticosterone may impair cognitive performance (Borcel et al., 2008; Dominguez et al., 2019; Oei et al., 2006). To minimise the potential for confounding stress and distraction effects, we allowed the birds considerable time to acclimate before testing (birds were in captivity for about 3 months prior to testing): at the conclusion of the acclimation period all birds appeared calm, readily approached the researchers, and in some instances could even be hand-fed. We further attempted to reduce stress and distraction by testing each bird in its home aviary. We did not attempt to quantify stress levels (e.g., in the form of circulating corticosterone) in captive birds. Capturing the birds in their large aviaries on a regular basis posed an unacceptable risk of harm or injury; sampling within a sufficiently short time window to exclude the possibility that elevated stress hormones were an artefact of capture was infeasible; and repeated capture risked negatively impacting the birds' trust in us as researchers, limiting our ability to test and interact.

In this study, we used two different detour tasks to investigate magpie self-control. The use of detour tasks as a measure of inhibitory control has recently been called into question (Kabadayi et al., 2018; van Horik et al., 2018). It has been suggested that performance on these tasks may better represent an animal's previous experience with transparent objects or their general exploratory behaviour, rather than an ability to suppress a response. Other studies also suggest birds may learn to go around a barrier instead of inhibiting a response (Kabadayi et al., 2018). To avoid this issue, all magpies were naïve to the transparent objects (cylinder and wall) when they were first presented. Magpies successfully detoured around both tasks *c*. 70% of the time, a score comparable to great tits (*P. major*), ravens (*Corvus corax*), and chimpanzees (*Pan troglodytes*), who all perform above 80% on similar tasks (Isaksson et al., 2018; Kabadayi et al., 2016). However, those animals had been pre-trained and were not naïve to their respective tasks. In addition, following the completion of the detour task, we presented the magpies with a control task to test whether they applied a learned behaviour. Magpies completed both control tasks, successfully eating the food through the hole 85% of the time. These two results provide further evidence that the inhibitory tasks challenged the bird's ability to inhibit a response, suggesting capacity for self-control.

Magpies did not learn the spatial memory task and performance on the spatial memory task was unaffected by both treatments and prior experience with the task. In fact, during the spatial task in both treatments, birds were no more successful at 24-h than at 48-h, selecting the correct well 26% of the time during both trials – equivalent to randomly selecting one of the four wells. Birds did improve performance in the olfaction trial, selecting the expected (i.e., previously baited) well 42.9% of the time, a 65% increase from the previous trial, which might indicate that they used olfactory cues or picked up on some other cue. The fact that our magpies did not learn the spatial memory task call into question whether we provided enough time for the birds to explore the task or if this task is suitable or relevant for magpies. While magpies have the ability to perform similar spatial memory tasks (Ashton et al., 2018; Connelly et al., 2022), there is little evidence that caching food is important to magpies. Spatial memory, often in the form of caching, is a behaviour in many wild bird species (Balda and Kamil, 1992; Lewis and Kamil, 2006). Unlike northern hemisphere species, birds in the southern hemisphere do not experience extreme winter conditions with low food availability, so caching is presumably an ecologically less relevant skill (Brodin and Urhan, 2014; Croston et al., 2017). Although we (F.C. and R.D.J.) have observed magpies caching food in the wild on multiple occasions, they seem to make little attempt to hide or camouflage food or retrieve food from a cache. Thus, until we learn more about the caching behaviour of magpies it remains unclear whether this specific spatial memory task performed in such a short timeframe quantifies an ecologically relevant cognitive ability of magpies. Constraints in animal facility space, ethical permissions and time constraints restricted the number of subjects in this experiment to seven birds for associative and reversal learning and nine birds for inhibitory control and spatial memory. These numbers are comparable to other cognitive studies on Australian magpies (Ashton et al., 2018: 15). These numbers are comparable to other cognitive studies on Australian magpies (*n* = 15, Ashton et al., 2018; *n* = 9, Johnsson et al., 2022a; *n* = 10, Mirville et al., 2016). Nevertheless, we recognise that the risk of committing a Type 2 Error increases with decreasing sample size and suggest that future studies could further explore the relationship between noise exposure and cognitive performance.

One link that still needs further exploring is that between sleep and cognitive performance. Sleep serves many functions, including diverse processes related to the maturation and maintenance of the central nervous system (Basner et al., 2013; Blumberg, 2015; Diekelmann and Born, 2010; Tononi and Cirelli, 2014; Xie et al., 2013). Indeed, waking performance depends on the amount of prior sleep in humans (Dawson and Reid, 1997; Van Dongen et al., 2003), honey bees (Klein et al., 2010), and birds (Aulsebrook et al., 2021), including Australian magpies (Johnsson, et al., 2022a). While acute noise exposure disrupts sleep in magpies (Connelly et al., 2020), understanding the effect of chronic noise on sleep in birds might help explain why cognitive performance in magpies appears to be robust to urban noise.

In conclusion, we found that exposure to an environmentally realistic anthropogenic noise playback does not impair cognitive performance in captive, wild-caught Australian magpies. These results are consistent with previous findings in Australian magpies tested in the wild (Connelly et al., 2022); further suggesting the resilience of a magpie's cognitive ability to urban noise. Magpies are urban exploiters (Connelly et al., 2022; Diquelou et al., 2016) that may benefit from the modification of natural landscapes by humans. Preferring fragmented forest habitats over densely forested areas (Kaplan, 2019), magpies thrive in city parks and sports fields, foraging not only on invertebrates on the ground, but on food left by people. While there is evidence that noise has a negative impact on magpie sleep (Connelly et al., 2020), magpies may have a relatively high tolerance for noise (Connelly et al., 2022) and this may help explain why negative impacts that have been documented in other species do not appear to manifest in Australian magpies (Kleist et al., 2018; Osbrink et al., 2021; Potvin, 2017; Salmón et al., 2016). As cityscapes continue to expand as a result of rapid human population growth, Australian magpies represent rare examples of urban exploiters that are nevertheless able to adapt and even thrive.

### Ethics approval

All methods were approved by the La Trobe University Animal Ethics Committee (AEC 18034). Birds were captured and released with permission from the Department of Environment, Land, Water and Planning (10008264) and the Australian Bird and Bat Banding Scheme (ABBBS authority number 1405). Following the completion of the study all birds were released: nine of the birds were seen roughly 1 month after release; five of the birds established territories within a year; and one bird was seen with a juvenile 2 years later, suggesting that any effects of captivity were short-lived.

## MATERIALS AND METHODS

### Animals and housing conditions

In January 2019, we captured 12 adult Australian magpies (six birds of each sex, based on plumage) (Kaplan, 2019) in the city of Melbourne, Australia, using a walk-in trap baited with grated cheese. All magpies came from non-breeding groups in urban parklands in the Melbourne suburb of Parkville. Sound levels across territories ranged from 40 to 65 dBA. The sites with the lowest levels of noise averaged between 40 and 45 dBA (equivalent to the sound of a library), while the loudest sites were between 60 and 65 dBA (equivalent to a busy restaurant). Using an NSRT Sound Level Logger (Convergence Instruments, Quebec, Canada) at 48 kHz sampling rates, we recorded sound levels (in dBA) at two or three geographically dispersed locations within each territory. Loggers were placed in trees approximately 5 m above the ground. Magpies were transported to an indoor animal holding facility at La Trobe University where they were housed in two experimental rooms with similar configurations (three females/males per room). Magpies were housed individually in aviaries 1.8 m high×1.8 m deep×0.9 m wide which were left uncovered, allowing the magpies to both see and hear one another. Each aviary contained three perches: two rectangular plank perches (15 cm wide), one 130 cm and the other 45 cm above the ground, and a dowel perch 45 cm above the floor. Each aviary contained two video cameras with infrared capabilities (one mounted above the 130 cm perch where the birds regularly slept; the other on the door of the aviary). The camera on the door focused on the front perch to record testing. Magpies were fed a mixture of minced meat and an insectivore mix (55 g; Wombaroo Food Products, Australia) once per day (at *c*. 0900 h); on test days, food was not provided until after the testing period (1130 h). Clean water was given daily in a large bowl, providing the magpies with a place to both drink and bathe. Aviary floors were covered by woodchips and, to provide enrichment, 15–20 mealworms were scattered daily throughout the woodchips, giving the magpies the opportunity to forage. Rooms were temperature-controlled (22±5°C) and insulated from all external light. Room lighting (153±18 lux) was set to a 12-h light (0600–1800 h), 12-h dark (1800–0600 h) photoperiod. Night lights (∼0.1 lux at sleeping perch) were placed in each room so that the magpies could move at night without harming themselves, and because true darkness in the wild is unrealistic for most birds (Aulsebrook et al., 2022).

Magpies were habituated to human experimenters via daily interactions over their first month in captivity. At the end of this period, the birds no longer reacted evasively to our presence, and in some instances could be fed by hand. Magpies also habituated to one another, with birds in both rooms singing together (Johnsson, et al., 2022a). To ensure good health, weight was monitored throughout captivity by training the birds to step on a scale. Magpies underwent surgery (approximately 2 months prior to this study) to implant sensors for recording brain activity for unrelated sleep experiments (Aulsebrook et al., 2020a,b; Connelly et al., 2020; Johnsson, et al., 2022a,b). To determine whether the surgical procedure altered cognitive performance, we presented the birds with a simple motor test (same test board as Spatial Memory) before and after surgery; there was no difference in the birds' ability to perform the task between the two time-points. This experiment was conducted approximately 3 months after initial capture.

### Experimental design

**Fig. 3.**
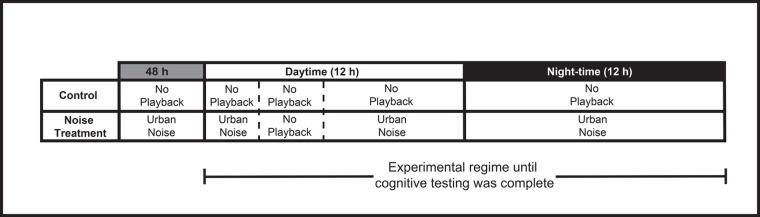
**Experimental design and testing regime for both the control and noise treatment.** Anthropogenic noise playback for the treatment began 48-h prior to the start of cognitive testing. Once testing began, noise playback was stopped for 3-h each day (0830–1130) to allow for relative quiet during testing.

We used a repeated measures design to compare performance on the cognitive test battery following exposure to and in the absence of anthropogenic noise (Fig. 3). While birds in one room received the noise treatment, birds in the other room received the (quiet) control. During the treatment round, the noise playback started 48 h before testing began. Birds were tested for 3 h each day (0830–1130 h), during which the playback was muted to prevent unwanted distraction due to the noise. Once all birds completed the test battery (completed all tests or ceased testing), the playback was switched off and birds had 7 days to recover. Following the week-long recovery, the treatment assigned to each room was switched and the experimental protocol commenced anew. Balancing the order in which treatments were presented across subjects was designed to limit any bias due to potential order effects. Overall, 9 out of 11 birds participated in this experiment (females: *n*=4; males=5), each bird testing in both the control (no noise) and noise treatments.

For the treatment, we broadcasted a recording of urban noise for the entire 12-h night and remnant 9-h day outside of the 3-h testing period. Noise for the playback was recorded over 1 week (Monday – Sunday) at a busy roadside located near the Melbourne Central Business District, providing a realistic city soundscape (Fig. S1). The playback was recorded using a Bioacoustic Audio Recorder (BARs, Frontier Labs; sampling rate: 44.1 kH gain, 20 dB); positioned 30 m from the nearest road. The time signatures of the playback and the experiment were synchronized, and sunrise/sunset times mismatched by less than 15 and 50 min, respectively, such that birds experienced realistic noises throughout each day/night period. An omni-directional speaker (Ultimate Ears, Boom 2) was placed in the centre of each room to broadcast the playback for the noise treatment. It should be noted that these speakers may not have accurately reproduced all low frequency sounds (<90 Hz) or vibrations. A sound level logger (Sound Level Meter Data Logger NSRT mk3; Convergence Instruments) was placed in each room, 3.5 m from the speaker to measure the sound levels represented by A-weighted decibels (dBA) from a single point in the room throughout each treatment. The average dBA level (hourly) of the playback over the testing period was 62 dBA (± 6.22), with noise levels ranging between 40 and 90 dBA throughout each 24-h period (Fig. S1); these were in the same range of the noise levels at the playback recoding location. The average sound level (62 dBA) was well below the safe threshold for humans and within the range of noise pollution found in metropolitan areas (Brown et al., 2015). In this way, the recording was an accurate representation of what urban magpies experience in the wild (Connelly et al., 2022). The background noise in the control treatment was 44 dBA (± 2.67), ranging between 42 and 63 dBA, and composed of constant low-level noise generated by ventilation and air-conditioning systems, and high-level noise associated with magpie carols and calls (Johnsson et al., 2022a). As intended, the soundscape of the room was louder when the recording of urban noise was played compared to when it was not (*t*=38.2, *P*<0.01).

### Cognitive testing and sample sizes

Prior to the start of the experiment, we used a motor skill task or ‘training board’ to train the magpies to interact with the test battery. The training board consisted of a wooden block (30 cm long×9 cm wide×4 cm high) with four equally spaced wells (3.0 cm deep, 3.3 cm diameter; Fig. 2). Covering the wells were four coloured plastic caps (two yellow and two grey). The plastic caps were held in place by a rubber band that looped around nails in the board. This set-up provided an axis on which the magpies could rotate the lids to access the wells. A few mealworms (3–5) were placed in each well as a reward. Magpies were presented with the training board for several days, until they successfully ate out of each well. The training board, as well as all other tasks, was positioned on the plank perch in the front of each aviary for testing.

We used a cognitive test battery to quantify differences in cognitive performance between noise treatments. Each magpie was tested daily during the 3-h testing period and presented with a task (typically) two times per day but dependent on motivation. Birds took on average 8±2 days to complete all tasks. Testing was considered finished once all birds in each treatment had either completed all tasks or had ceased testing (i.e. 3 days without interacting with a task). Magpies were tested in their respective aviaries with curtains hung between cages to ensure testing occurred in isolation.

The cognitive test battery consisted of four main tasks (see Test battery for details). Tasks were always presented in the same order, and each tested a well-defined cognitive function of presumed ecological relevance:

(1) Associative learning: The ability to acquire knowledge through repeated experiences (Morand-Ferron et al., 2016).
(2) Reversal learning: The ability to learn a new rule, a measure of cognitive flexibility (Bond et al., 2007).
(3) Inhibitory control: A measure of self-control based on the inhibition of immediate responses (Isaksson et al., 2018; Kabadayi et al., 2016; 2018).
(4) Spatial memory: The ability to remember the location of resources or threats (Emery, 2006).

Tasks 2 and 3 might be indicative of executive functions (top-down cognitive processes exerting control over information processing; Bobrowicz and Greiff, 2022). A new task could begin immediately after the previous task was completed, with the exception of reversal learning, which always started 1 day after the completion of the associative learning task. The criterion for success varied between tasks, but in all tasks the total number of trials required to fulfil the success criterion acted as the test score. Therefore, fewer trials taken to reach their task-specific success criterion indicated a bird performed ‘better’ (see Test battery for details). Tasks only began when a magpie was motivated enough to approach and eat a mealworm. The training board was functionally identical to the associative learning, reversal learning, and spatial memory tasks, such that birds did not need to be further trained to operate those tests. Sample sizes varied among tests – associative learning: *n*=7; reversal learning: *n*=7; inhibitory control: *n*=9; spatial memory: *n*=9 owing to some birds not interacting with, or completing, the task.

Because birds were presented with each task twice in the repeated measures design, minor aspects of the task were modified between the treatments to introduce novelty and test for genuine learning while allowing the pair-wise comparison of scores. For instance, in the associative learning task, the colour of the caps covering the wells was blue for the noise treatment and purple for the control, thus providing a comparable test, but with a novel colour.

### Test battery

#### Associative learning

We used a colour-discrimination task to test how quickly magpies could learn to associate a novel-coloured cap with a reward. The foraging grid (19 cm long×9 cm wide×4 cm high) contained two wells (3.0 cm deep, 3.3 cm diameter; Fig. 2). Each well was covered with a cap of a different shade of the same colour (i.e. navy and sky blue; violet and lilac purple). Shades of the same colour were used instead of different colours to minimize any potential effects of colour preference. One well contained a chilled (non-moving) mealworm reward; the other well was empty. The colour of the rewarded cap was randomized, but once selected, was maintained throughout the entire testing round (treatment). In the first trial, magpies were allowed to peck both caps in order to find the reward under the correct cap. In all consecutive trials, pecking the incorrect cap cued the removal of the board for one minute. Birds had a maximum of 1 min to interact with the board before it was removed from the testing area. Testing resumed only when birds showed interest in interacting with the test boards again (i.e. by returning to the ‘testing area’ or lower perch). The position of the rewarded cap (left or right) was pseudo-randomized, meaning we avoided having the reward on the same side for more than three consecutive trials. The task was considered complete when the subject pecked the correct cap (obtaining the reward) in 10 out of 12 consecutive trials; a significant deviation from random binomial probability (binomial test: *P*=0.04; same criterion as in Ashton et al., 2018; Connelly et al., 2022; Johnsson et al., 2022a). If the bird did not reach the criterion in a single day the score would carry over to the next day and testing would continue. The total number of trials required to reach this criterion (including the final 12 trials) represented the associative learning score. To prevent olfactory cues, both wells were wiped with chilled mealworms prior to the start of testing each day (similar to Ashton et al., 2018). To maintain novelty, the colour of the caps was different for each treatment.

#### Reversal learning

The reversal learning task tested cognitive flexibility, quantifying the number of trials required for a magpie to dissociate the previous association and learn a new rule (Fig. 2). Reversal learning used the same board as associative learning; however, the shade of the of rewarded cap was switched (i.e. if the reward cap was navy for the association learning task, it would be sky-blue for the reversal learning task). The experimental protocol and completion criterion were the same as for the associative learning task.

##### Inhibitory control

There were two inhibitory control tasks, and both quantified the magpies' ability to inhibit predominant responses, and efficiently navigate around an obstacle to achieve a goal (in this case, to obtain a food reward). Here, the predominant response would be for the bird to try to retrieve a reward by pecking at a transparent plastic barrier placed in front of a food reward. We presented magpies with two such ‘detour reaching’ tasks that were visually distinct, yet functionally similar. In the first task, a food reward (1–3 mealworms) was placed inside a transparent open-ended cylinder (13 cm long×5 cm diameter) mounted on a small wooden block (13 cm long×6.5 cm wide×5 cm high) (Fig. 2). The cylinder was presented perpendicular to the subjects' body axis (i.e. open ends were positioned out of the magpies' eyeline), to see if they could solve the challenge of accessing the reward. In the second inhibitory task, the cylinder was replaced with a transparent plastic wall (34 cm wide×13 cm high) mounted vertically onto a wooden block (24 cm long×9 cm wide×4 cm high; Fig. 2). The same reward was placed behind the wall.

For both tasks, when first presented, the subject was allowed to explore the test board and find their reward. Each trial thereafter counted towards their overall score. A trial was considered successful if the subject did not peck the closed side of the cylinder or the plastic wall, and instead walked to the open ends of the cylinder, or around the wall, to obtain the mealworm. If the bird pecked the cylinder or wall, an incorrect score was tallied, and the test was removed from the testing area for 1 min. The task was considered complete when the subject correctly detoured (without pecking) around the task to obtain the reward three consecutive times. Following and to be consistent with established protocols (Ashton et al., 2018; Connelly et al., 2022) magpies were allowed a maximum of 10 trials per day on the detour tasks, and trials were conducted in 1-min intervals. Test results for individuals on the two detour tasks were not statistically different (t=0.91, *P*=0.38) and we therefore tallied the number of trials taken to pass both these tasks into a single ‘inhibitory score’ per individual for the analysis.

After completion of the inhibitory testing, we presented the magpies with a ‘control’ task to determine if the birds were truly inhibiting the urge to peck and not simply learning to take a route around the barrier (an associative learning function). These tasks consisted of the same cylinder or wall (Fig. 2), but with a hole cut out of the centre allowing the magpies to access the reward without detouring around the obstacle. A magpie successfully completed a trial if they retrieved the reward through the cut hole instead of navigating around the obstacle. The success criterion was the same as for the inhibitory tasks.

##### Spatial memory

The spatial memory task was used to determine a magpie's ability to store location-based information for later retrieval. The task consisted of a wooden board (45 cm long×9 cm wide×4 cm high) containing an array of 1×4 wells (3.0 cm deep, 3.3 cm diameter; Fig. 2). This was modified from 2×4×2 well system used by previous studies (Ashton et al., 2018; Connelly et al., 2022) to better fit the physical constraints of the captive testing environment. Each well was covered with a lime green cap and used the same flipping mechanism as the associative and reversal learning tasks. Wells were numbered (1–4) from left to right. Each individual was randomly assigned a well number and that well contained the food reward for the entirety of the test; the well was always baited out of site. Magpies were presented with the test board five times over 3 days: twice on the first day, once on the second, and twice on the third (as per Connelly et al., 2022). Each testing ‘cycle’ was deemed complete when the magpie found the rewarded well. The first cycle (or baseline) had the magpie presented with the board to find the reward. Once found, the board was removed. After 5 min the same well was rebaited, and the second cycle (or training) began, following the same protocol. The third and fourth cycles were presented in an identical way, 24 h and 48 h later, respectively. The total number of trials required to find the food reward in the third and fourth trials determined a magpie's spatial memory score. The well assigned with the food reward was different for each bird in each treatment. A final trial (or ‘smell’ trial) was performed 5 min after the fourth trial. For the smell trial, the board was rotated 180 degrees, and no reward was placed in the wells. The foraging grid appeared identical to the subject, but the previously baited well was on the opposite side of the test board. This test was conducted to assess whether magpies might be using olfactory cues to determine the location of the baited well.

### Statistical analysis

All analyses were conducted in the statistical environment R version 3.5.2 (R Development Core Team 2018). Using the R package *lme4*, we applied generalized linear models (GLMM) with a Poisson error structure and logarithmic link to investigate the factors affecting performance on all tasks. The GLMM contained all probable explanatory values: treatment (noise or control), treatment order (noise first or second), test order (first or second trial), sex, and the interaction of the test type (associative learning, reversal learning, inhibitory control, spatial memory) and treatment (Table 1). Random effects were bird identity and housing room (one or two). Paired *t*-tests were used to compare performances on individual tests.

## Supplementary Material

10.1242/biolopen.060535_sup1 Supplementary information
