# Supplementary material for: Experimental playback of urban noise does not affect cognitive performance in captive Australian magpies
Source: Biol Open. 2024 Aug 14;13(8):bio060535. doi: 10.1242/bio.060535 (PMC11340814; doi:10.1242/bio.060535)
Supplement: Supplementary information [file biolopen-13-060535-s1.pdf]

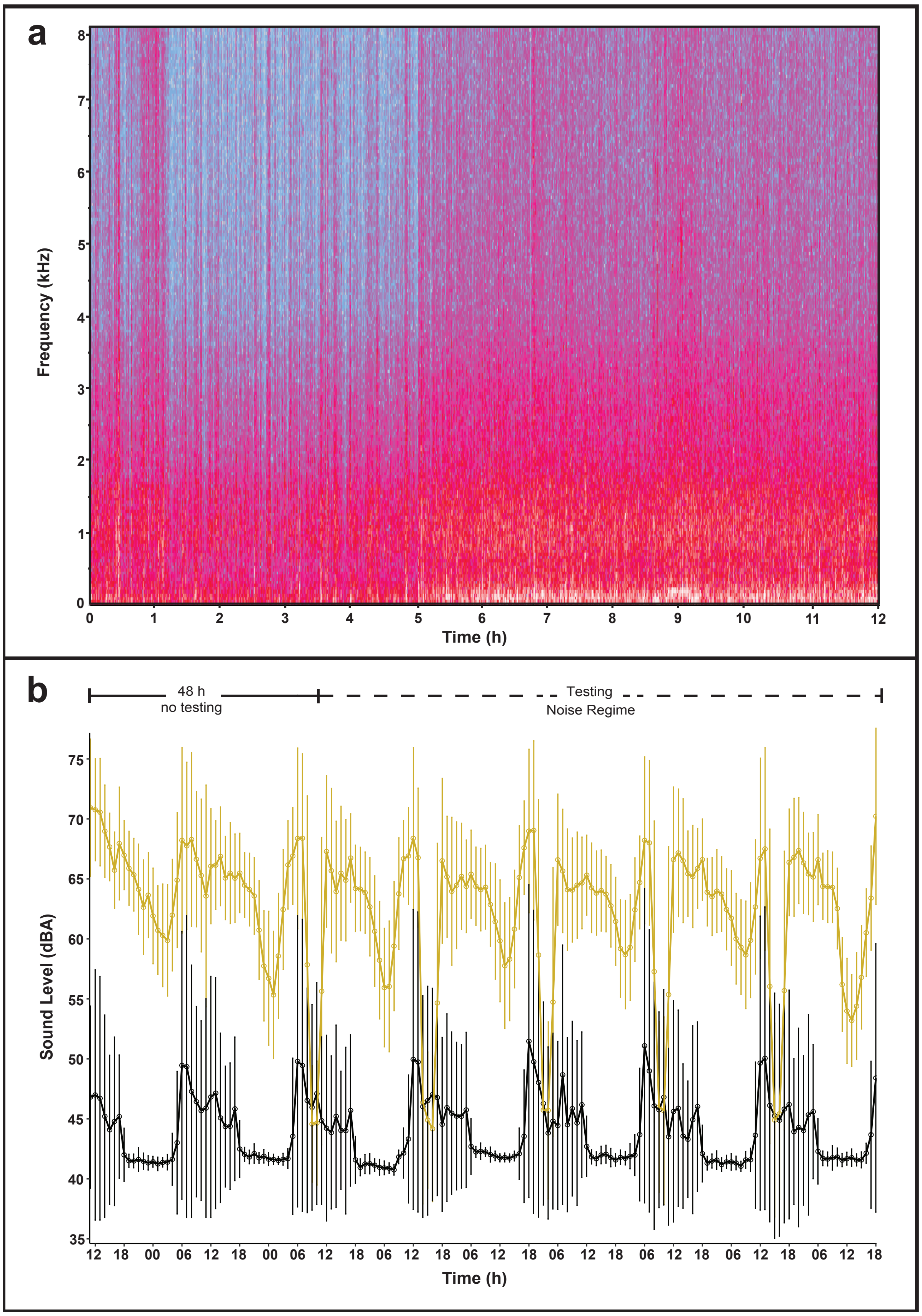
a
Frequency (kHz)
Time (h)
b
48 h
no testing
Testing
Noise Regime
Sound Level (dBA)
Time (h)

Fig. S1.

Table S1. Raw data on the performance of each bird on the test battery (colour association, reversal learning, inhibitory control, and spatial memory). Each row describes a single birds performance on the test battery in each treatment (two rows per bird). Columns detail the birds' name, the treatment it was exposed to (control or high noise level), the test order (if this was the birds first or second attempt at the test battery), the order the noise was presented (if the bird was exposed to the noise treatment on its first attempt at the test battery or second), and the number of trials it took to complete each cognitive task within the test battery.

Bird Name	Treatment	Test Order	Order Noise Presented	Colour Association	Reversal Learning	Inhibitory Control	Spatial Memory
Cox	control	first	second	-	-	8	-
Swan	control	first	second	47	58	18	3
Darcy	control	second	first	35	19	25	2
De Goey	control	first	second	22	33	18	3
Grundy	control	second	first	10	58	6	6
Pendlebury	control	first	second	-	-	9	10
Sidebottom	control	second	first	20	16	12	7
Taylor	control	second	first	-	-	-	10
Treloar	control	first	second	60	18	12	6
Varcoe	control	second	first	60	97	6	7
Cox	high	second	second	-	-	9	-
Swan	high	second	second	20	22	14	4

Darcy	high	first	first	41	19	12	4
De Goey	high	second	second	10	85	10	5
Grundy	high	first	first	36	17	9	2
Pendlebury	high	second	second	-	-	7	6
Sidebottom	high	first	first	61	82	45	12
Taylor	high	first	first	-	-	-	2
Treloar	high	second	second	23	29	6	2
Varcoe	high	first	first	61	72	15	8

Biology Open • Supplementary information
